# New work situations call for familiar work design methods: Effects of task rotation and how they are mediated in a technology-supported workplace

**DOI:** 10.3389/fpsyg.2022.935952

**Published:** 2022-10-12

**Authors:** Lisa Mlekus, Janine Lehmann, Günter W. Maier

**Affiliations:** ^1^Department of Psychology, Bielefeld University, Bielefeld, Germany; ^2^Research Institute for Cognition and Robotics – CoR-Lab, Bielefeld University, Bielefeld, Germany

**Keywords:** work design, task rotation, job rotation, digital assistance system, experiment, job satisfaction, affect, performance

## Abstract

New digital assistive technologies strive to alleviate the completion of work tasks but thereby often threaten to make jobs increasingly monotonous. To counteract jobs becoming more and more monotonous, task rotation might be an appropriate technology feature. However, it is uncertain whether task rotation has unique positive effects, why it works, and whether there are any boundary conditions. To investigate this, we conducted two experimental vignette studies. In Study 1 (*N*_1_ = 135), we drew on the job characteristics model and self-determination theory to examine perceived task variety, skill variety, and task identity, and expected satisfaction of the need for competence as mediators of the effect of task rotation on anticipated employee attitudes (job satisfaction, intrinsic work motivation), behavior (subjective performance), and well-being (positive and negative affect). The investigated vignette described a job where a digital assistance system either indicated the task rotation or only supported work steps. Regression analyses showed direct effects of task rotation on expected job satisfaction, intrinsic motivation, and positive affect. There were indirect effects of task rotation on all outcomes except expected negative affect. We used Study 2 (*N*_2_ = 159) as an exact replication of Study 1. Additionally, to investigate the boundary conditions of task rotation effects, we drew on person-job fit theory and investigated openness to experience as a moderator of the effects of task and skill variety on the outcomes. Regression analyses showed direct effects of task rotation on expected job satisfaction, subjective performance, and positive affect. There were indirect effects of task rotation on all outcomes except expected negative affect and intrinsic motivation. Thus, the results of Study 1 could only be partly replicated. Openness to experience did not moderate the effects of task and skill variety on the outcomes. The results support the relevance of task rotation as a technology feature and indicate that rotations should offer especially skill variety and task identity, as these were the strongest mediators in our studies.

## Introduction

The currently increasing adoption of advanced technologies in the workplace affects the way work is designed and how employees experience their work (Montealegre and Cascio, 2017; Parker and Grote, 2020; Wang et al., 2020). Depending on the concrete technology and its features, there are various possibilities. A basic distinction can be made between technologies that either substitute for or that complement workers in performing specific work tasks (Autor et al., 2003). An example for the substitution of work tasks are industrial welding robots, which perform monotonous and dangerous tasks previously done by employees (e.g., Efimov et al., 2019). The adoption of these technologies can provide relief for the employees, allows them to perform more meaningful tasks, and reduces human errors. Technologies that complement employees are, for example, digital assistance systems. These provide situational support in accordance with work progress, so employees need less knowledge of processes (Reinhart and Patron, 2003; Gensler et al., 2022). The adoption of these technologies reduces the need for initial training but can make the job more monotonous because the employee only needs to perform the indicated tasks. In this latter case, which is the focus of the present study, researchers and practitioners are faced with the challenge of designing workplaces where technologies promote, rather than threaten, motivating and fulfilling work (Montealegre and Cascio, 2017). Hackman and Oldham (1976), and later also Morgeson and colleagues (Morgeson and Humphrey, 2006; Morgeson et al., 2012), illustrate in their work design models that key outcomes of fulfilling work are positive employee attitudes (e.g., job satisfaction, internal work motivation), improved behavior (e.g., performance), developed cognitions (e.g., learning), and enhanced well-being (e.g., affect). These core outcomes of work design have also been emphasized by a more recent systematic review (Knight and Parker, 2019). Parker and Grote (2020) also stressed in their literature review on the interplay of work design and technology that technology has the potential to result in both: an upgrading and upskilling of work when technology takes over the “dull, dirty, and dangerous tasks” (p. 15), or technology “creates problems for motivation and performance” (pp. 15–16) when the resulting job is highly specialized and standardized. Thus, the question arises how good work design can be considered in technology design to avoid monotonous, unfulfilling jobs.

The combined consideration of technology and its social context is the central tenet of both sociomateriality and sociotechnical systems theory (Pasmore et al., 1982; Orlikowski, 2007; Leonardi, 2012). While sociomateriality stems from the field of management information systems, and sociotechnical systems theory originates from the field of work design, both theories are closely related in that they argue that studying technology without its context—or vice versa—results in an incomplete view (Cherns, 1987; Orlikowski and Scott, 2008). In a recent review of technology integration in organizational psychology and organizational behavior (OP/OB), Landers and Marin (2021) discuss strategies how OP/OB researchers currently consider technology in their studies and evaluate the usefulness of each approach. They conclude that the most future-proof approach is to consider technologies in terms of their specific features and design characteristics (e.g., pedestrian detection in autonomous cars), as opposed to investigating a certain technology as a whole and comparing it with control groups without the technology (e.g., autonomous driving vs. human-controlled driving). The authors argue that the latter approach would produce outdated knowledge as soon as the technology receives an update because the effects found in a previous study might have been caused by a feature that no longer exists after the update.

When considering digital assistance systems, and their risk of creating simpler and more monotonous work, there is a classic work design method that could be adopted to reduce these risks: task rotation. Task rotation is a work design technique in which employees shift periodically and in a planned manner between a range of tasks in their workplace (Jones and James, 2018). It makes jobs richer in variety, and previous studies found that it was positively associated with a range of positive outcomes, such as job satisfaction, work motivation, performance, and psychological health (cf. for a recent meta-analysis, Mlekus and Maier, 2021). Such effects have, however, not yet been studied in the context of digital assistance systems. Our manuscript aims to fill this gap because of the great importance of context-sensitive research. Context refers to opportunities or constraints innate in a situation or environment that can either affect work design characteristics or interact with these characteristics and with individual variables to affect outcomes (Johns, 2006, 2018; Morgeson et al., 2010). In his much-cited essay, Johns (2006) illustrated that research in organizational behavior lacked a consideration of the context in which a study was conducted. He argued that context could cause variations between studies on the same subject, and that its consideration is therefore necessary to correctly interpret study findings and to derive more fitting applications of research. Additionally, there are further open questions about task rotation that existing studies have not answered: To better design and investigate task rotation interventions, practitioners and researchers need to know whether existing findings can actually be attributed to unique, causal effects of task rotation, why task rotation works, and whether there are circumstances that might enhance or decrease its effect. As technologies that plan a rotation are, to our knowledge, not yet widely used in organizations, our aim in the present study was to investigate the technology feature task rotation and the corresponding expectations and perceptions individuals have regarding task rotation in a prospective job.

The first question is whether task rotation has a unique, causal effect on the expected work design outcomes of job satisfaction, intrinsic work motivation, subjective performance, and affect. To answer this, studies would need to have an experimental or quasi-experimental design. Only in a (quasi-)experimental setting, the relationship between an independent variable and a dependent variable can be attributed to a causal effect. However, the majority of (quasi-)experimental studies have only investigated the effects of task rotation on ergonomic factors and physical health (for a literature review see, e.g., Padula et al., 2017). To our knowledge, there are only three studies that have analyzed psychological outcomes of task rotation in a (quasi-)experiment (Luger et al., 2016; Comper et al., 2017; Jones and James, 2018), and two of those focused on the more general outcome psychological quality of life. It is important to investigate the unique, causal effects of task rotation because results from correlative studies might only be the result of other unknown or uncontrolled variables. For example, companies that adopt task rotation could also be more likely to allow flexible working hours. Thus, task rotation and flexible working hours might be confounded.

The second question is why task rotation is effective. So far, studies that investigated task rotation have rarely based their assumptions on psychological theories. Instead, most authors derived their hypotheses from prior evidence or practitioner-oriented management approaches, such as the concept of high-performance work systems (e.g., Selden et al., 2013; Avgoustaki, 2016). Exceptions are, for example, Hsieh and Chao (2004), who have pointed to the job characteristics model (JCM), and Cruz and Pil (2011), who based their hypotheses on the job demands-control model. The theoretical basis of this manuscript are the JCM (Hackman and Oldham, 1976) and self-determination theory (e.g., Deci and Ryan, 2014), combined with Parker et al.’s (2017a) framework of work design influences. The JCM is “the most influential model of work design” (Parker et al., 2017b, p. 407), but it is also limited. More specifically, it does not describe the context that creates the existence of certain work characteristics. Instead, this has been done by Parker et al.’s (2017b) framework, and is realized in this manuscript by including task rotation as the work context. Thus, our manuscript contributes to task rotation knowledge, because no study has tested whether the perception of certain work characteristics mediates the effects of task rotation on employee-related outcomes. This is problematic because there could also be other explanations for the beneficial effects of task rotation, such as that it improves physical health, which in turn benefits employee motivation and performance. Additionally, self-determination theory offers a promising addition to the JCM, as it provides alternative mediating mechanisms.

The third question is whether task rotation is universally effective or whether there are individual differences that might decrease its effects on the employees. Based on person-job fit theory (Edwards, 1991), one could expect that differences in personality characteristics could alter the effects of work design. The theory proposes that compatibility between an employee’s needs, desires, or preferences and their job results in more positive job attitudes and behavior, such as job satisfaction and performance (Edwards and Shipp, 2007). Studies found, for example, that self-competence affected the relationship between job enlargement and job crafting (Berdicchia et al., 2016), and proactive personality altered the associations between high-performance work systems, such as flexible job design, and, for instance, task performance (Zhang et al., 2019). Regarding task rotation, we expected that employees with higher openness to experience might perceive rotation as more beneficial. Jobs with task rotation are characterized by changing work environments, diverse tasks, and a certain level of uncertainty, and thus match the preference for variety, a need for change, and an aversion to routines that people with high openness have (De Jong et al., 2001). We therefore investigated whether openness to experience had an influence on the effects of work characteristics that are related to task rotation on expected employee attitudes, behavior, and well-being.

In this study, we contribute to the understanding of task rotation as a technological feature by investigating answers to these pressing questions through two consecutive studies. We employed experimental vignettes as a means to investigate participants’ perceptions of work characteristics and expectations of their reactions in terms of attitudes, behavior, and affect. The vignette methodology was suitable because we wanted to gain insights on how people evaluate prospective work design. In our first study, we investigated four mediators of the effects of task rotation on psychological outcomes. By analyzing all possible mediators simultaneously, we sought to identify the strongest mediator. Additionally, as we conducted an experiment, we were able to analyze the unique, causal effects of task rotation. The aim of our second study was two-fold. First, we added the moderator openness to experience and analyzed whether it might influence the effects of perceived work characteristics, namely task variety and skill variety, on the investigated outcomes. Second, by keeping everything else the same as in the first study, we wanted to test the reproducibility of the findings of the first study to ensure that they were not only due to chance. As Kepes and McDaniel (2013) pointed out, exact replications are one way to be more confident about the effects found in a study. Both studies had sample sizes that were large enough to detect significant medium-sized main effects.

## Task rotation as a technology feature

In modern workplaces, work design is increasingly intertwined with the adoption of advanced technologies. Current technological advancements allow organizations to integrate intelligent systems that can collect and process information about their environment via sensors and thus react in real-time to problems and requests (Cascio and Montealegre, 2016). Digital assistance systems are one class of such technologies and include, for example, augmented reality glasses or workspace-integrated displays that instruct employees how to perform each step of a task (Wang et al., 2016; Yang and Plewe, 2016; Paruzel et al., 2020a). The key feature of digital assistance systems is that they provide the worker with relevant information in a given situation. This information may include, for example, step-by-step instructions or remarks on special cases (Reinhart and Patron, 2003). This makes digital assistance systems especially useful for workplaces where employees need to be able to perform the necessary work steps quickly.

Engineers who design technological systems primarily aim to ensure productivity, workplace safety, or the reduction of human errors (Djuric et al., 2016; Hold et al., 2017), and rarely consider the psychological criteria of work design. In the long term, workplaces with digital assistance systems might therefore be perceived as simple and undemanding because employees no longer need the knowledge and/or skills that were necessary to perform the job without assistance. Such unchallenging jobs can result in a decrease in job satisfaction and performance, among other things (Humphrey et al., 2007). Task rotation could be an adequate means to increase the variety in such repetitive jobs and thus counteract possible negative effects. Task rotation describes the process of having employees alternate between highly physically demanding tasks or repetitive tasks to tasks that pose less physical strain or that provide more variety. The aim is to enhance the motivating potential of the job, and reduce physiological, psychological, or biological strain (Spitzmüller and Sady, 2007). The technological possibilities even allow task rotation to be integrated as a feature in digital assistance systems so that the system plans and indicates the rotation cycles. There is much research on creating algorithms for task rotation scheduling that aim at reducing monotony and boredom during task rotation (e.g., Bhadury and Radovilsky, 2006; Azizi et al., 2010).

Several studies have investigated job and task rotation (job rotation describes the rotation between whole jobs instead of tasks) and how they are related to positive employee or organizational outcomes (for a recent meta-analysis, see Mlekus and Maier, 2021). These relationships can be theoretically explained by Parker et al.’s (2017a) framework of work design influences and the JCM (Hackman and Oldham, 1976). Parker et al. (2017a) describe in their model several aspects that can be regarded as antecedents of work design, such as individual influences, managers’ motivation or organizational influences. One facet of organizational influences that is discussed in their framework, are human resources practices – such as task rotation. The authors describe that these can have direct and indirect effects on work design characteristics, for example, the autonomy, complexity or workload at a job. Numerous work design characteristics, in turn, are known to be associated with a range of positive employee outcomes (e.g., Humphrey et al., 2007), as was first theoretically described in the JCM. Hackman and Oldham (1976) assumed in their model that there were five job characteristics that prompted so-called critical psychological states, which in turn led to beneficial personal and work outcomes. The concrete job characteristics related to task rotation are discussed in more detail in the section on mediating mechanisms of task rotation effects.

Empirical evidence supports the expected association between task rotation and positive employee outcomes. In jobs with task rotation, employees were more satisfied with their job and had greater work motivation (e.g., Muramatsu et al., 1982; Jeon et al., 2016). Rotation was also associated with greater psychological well-being (Jones and James, 2018). Jones and James experimentally compared workers in an underground coal mine with and without task rotation. Those with task rotation had significantly better ratings on the psychological dimension of the WHOQOL questionnaire (World Health Organization, 1998), which includes the facets “positive feelings” and “negative feelings.” There are also organizational benefits to rotation. When employees switched between different workstations, they were more flexible (Sawhney, 2013), which means that they could be assigned spontaneously to jobs with a temporary high workload to prevent downtime and thus increase the organization’s performance. Studies also found a positive association between rotation and individual worker’s performance (Campion et al., 1994; Khan et al., 2014). It thus appears that both the employees and the organization benefit from task rotation.

What these studies have in common, however, is that the rotation was planned by a supervisor or the employees themselves. Task rotation has not yet been investigated in a workplace where a technology is used to plan the rotations, although it is a likely work scenario in the near future (White et al., 2020). There might be differences because technology could increase the feeling of being monitored, or because technology might plan the rotations based on criteria different from that a supervisor would use, among other things. On the other hand, it is possible that the person or algorithm planning the rotation does not play a central role, and that rotation is always perceived better than no rotation. This latter assumption fits our theoretical considerations best, as these are based on the way the work is designed and make no assumptions about the person (or algorithm) designing the work. Thus, before examining any of the underlying mechanisms of task rotation, we investigated whether the relationships from previous, mainly correlative, studies could also be attributed to causal effects, and expected in a technology-supported workplace.

*Hypothesis 1*: Task rotation has a positive effect on expected (a) job satisfaction, (b) intrinsic work motivation, (c) subjective performance, and (d) positive affect, as well as a negative effect on expected (e) negative affect.

## Mediating mechanisms of task rotation effects

**FIGURE 1:**
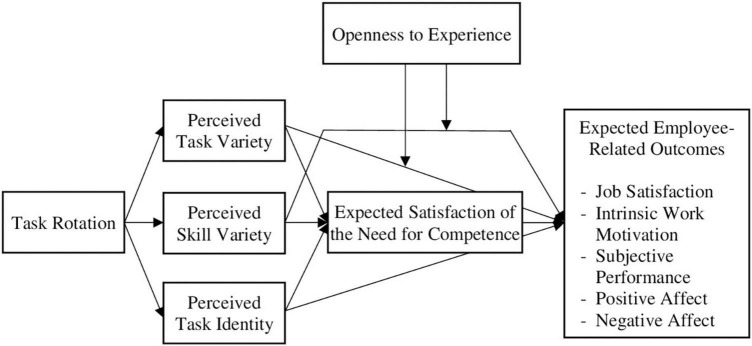
Openness to experience was only investigated in Study 2.

Although the outcomes of task rotation have been investigated in various studies, there is still great uncertainty on the question of why task rotation might have beneficial effects. One explanation could be that rotation between different tasks improves the employees’ physical health because the tasks stress different body regions and thus leave time for recovery (Mathiassen, 2006). Studies found that musculoskeletal complaints were associated with a range of employee responses, such as job satisfaction (Sobeih et al., 2006) and work motivation (Størseth, 2006). Another reason why task rotation might affect employee attitudes, behavior, or affect is that it could be perceived as a reward or a privilege, comparable to flexible working hours. When given free choice, the vast majority of employees in a study by Jeon et al. (2016) preferred some type of task rotation to no rotation. Another explanation, which we focus on in this manuscript, is that task rotation has an impact on employee responses because it enhances certain work characteristics that satisfy basic human needs. In the following, we derive the assumption that the effects of task rotation on anticipated employee-related outcomes are mediated by the parallel mediators perceived task variety, skill variety, and task identity, and the serial mediator expected satisfaction of the need for competence. The conceptual model is displayed in Figure 1.

### Task variety, skill variety, and task identity as mediators

We first drew on the JCM by Hackman and Oldham (1976) to explain why task rotation would have positive anticipated effects on employees. The model posits that there are five work characteristics that contribute to employee motivation: skill variety, task identity, task significance, autonomy, and feedback. Morgeson and Humphrey (2006) identified further factors and extended the JCM into a work design framework with task, knowledge, social, and contextual characteristics. In their meta-analysis, Humphrey et al. (2007) found evidence supporting the extended framework and also reported effects on other employee-related outcomes besides motivation, such as performance, stress, and job satisfaction.

More recently, Parker et al. (2017a) have reviewed possible antecedents of work design and stated that one of them were human resource practices, such as task rotation. The JCM and its extension, the work design framework, provide an explanation *why* task rotation might have beneficial effects on employees, thus give indications on the mediating mechanisms. A job where the employee alternates between different tasks does not necessarily provide all motivating work characteristics. Compared to a job without task rotation, however, it offers a greater variety of tasks (i.e., task variety), possibly requires more diverse skills (i.e., skill variety), and is more likely to consist of tasks that make up a complete work process (i.e., task identity). In the following, we will describe how task rotation may be indirectly associated with positive employee-related effects, mediated by task variety, skill variety, and task identity. Each section establishes how task rotation and the respective work characteristic are associated, followed by indications that the work characteristic is in turn associated with positive employee-related effects.

*Task variety* refers to the extent to which a job requires the employee to perform a diverse set of tasks. It is one of the characteristics that were not part of the original JCM but were added by Morgeson and Humphrey (2006). They argued that it is conceptually different from skill variety because it indicates the performance of multiple tasks as opposed to the use of multiple skills. Task rotation should increase the perceived task variety because the main aim of task rotation interventions is to increase the number of different tasks for the employee (e.g., Kuijer et al., 2005; Horton et al., 2015). Task rotation can thus be distinguished from interventions where employees perform a single task that is merely interrupted by rest breaks (Luger et al., 2014). Greater task variety is in turn associated with positive employee-related outcomes. Meta-analytic correlates of task variety included, for example, job satisfaction and subjective job performance (Humphrey et al., 2007). When the cited meta-analysis was conducted, there was only limited empirical research analyzing the impact of task variety on other work design outcomes. More recent studies found, for example, significant positive relationships between task variety and vigor (i.e., willingness to invest effort and persist when facing challenges) and dedication (i.e., a strong identification with one’s job; Salanova and Schaufeli, 2008), which could be indicators for an association between task variety and intrinsic work motivation. Also, there are studies that found that the employees’ affect was more positive when a job provided more task variety (Gillet et al., 2015; Wu and Wang, 2015). Thus, we assumed that the effect of task rotation on the expected positive employee-related outcomes could be partly explained by the fact that a job with task rotation provides more task variety than a job without task rotation.

*Skill variety* reflects the degree to which an individual needs to use a multitude of skills to perform a job successfully (Hackman and Oldham, 1976). It can be assumed that task rotation affects the perceived skill variety in a job because a job with multiple tasks is likely to require more different skills than a job with only one task. For instance, in the study by Kuijer et al. (2005), the employees at a refuse collecting department rotated between truck driving and refuse collecting. One task required the employees to know how to drive a heavy vehicle, the other required knowledge about recyclable materials and the skill of using the lifting mechanism of the refuse truck. It is theorized that jobs that require a variety of skills are perceived as more challenging, which increases the experienced meaningfulness of the job and engages employees (Hackman and Oldham, 1976). Meta-analytic results showed positive associations between skill variety and job involvement, internal work motivation, and job satisfaction (Humphrey et al., 2007). Additionally, primary studies also found positive relationships between skill variety and performance (Moran et al., 2012) and positive affect, and a negative relationship between skill variety and negative affect (Munz et al., 1996). In conclusion, we assumed that the effect of task rotation on the expected positive employee-related outcomes could be partly explained by the fact that a job with task rotation also provides more skill variety than a job without task rotation.

Lastly, *task identity* describes the extent to which an employee is required to perform a whole work process from start to finish with a visible outcome (Hackman and Oldham, 1976). In most cases, jobs with task rotation should be perceived as more complete compared to jobs with a single task. For instance, in the study by Balogh et al. (2016), task rotation meant that employees alternated between several tasks in a supermarket (retrieving goods from the storeroom, stocking shelves, attending to customers at the counter, doing cashier work). They were thus able to experience all services necessary for a supermarket to serve its customers. According to the JCM, the possibility to complete a work process from start to finish elicits pride in employees, which then serves as a motivator (Hackman and Oldham, 1976). Meta-analytic evidence found positive correlations between task identity and, for example, job satisfaction, internal work motivation, subjective performance, and reduced stress (Humphrey et al., 2007). Munz et al. (1996) also found a positive relationship between task identity and positive affect, and a negative relationship with negative affect. Consequently, we assumed that the effect of task rotation on the expected positive employee-related outcomes could be partly explained by the fact that a job with task rotation also provides more task identity than a job without task rotation.

### Satisfaction of the need for competence as a serial mediator

The JCM states that the relationships between skill variety and task identity and employee-related outcomes are mediated by experienced meaningfulness (i.e., the degree to which the employee feels that the job is important and has value; Hackman and Oldham, 1976). Humphrey et al. (2007) found partial support for this assumption in their meta-analysis: Experienced meaningfulness mediated the relationships between the two work characteristics and job satisfaction and internal work motivation, but only partially mediated the relationship with subjective performance, and other outcomes could not be addressed at all due to a lack of primary studies. It was thus reasonable to examine an alternative mechanism as a serial mediator that might explain the effects of task rotation, via perceived work characteristics, on anticipated employee-related outcomes.

Gagné and Panaccio (2014) suggested integrating self-determination theory within the JCM by using the satisfaction of basic human needs as mediators between work characteristics and motivation. Self-determination theory is a macro theory of human motivation. One tenet of the theory is that there are three basic psychological needs innate in every human being: the needs for autonomy, relatedness, and competence. The satisfaction of these needs is supposed to be the prerequisite for autonomous motivation, which means that an activity is done out of interest and enjoyment or because it is important to one’s values (Deci and Ryan, 2014).

Although self-determination theory states that all three needs should be satisfied, our focus was on the satisfaction of the need for competence, as we assumed that it could explain why task variety, skill variety, and task identity had positive effects on employees. As de Gieter et al. (2018) pointed out, depending on the research aim, it is legitimate to concentrate on only one or two of the basic needs. The need for competence is defined as the need to feel effective in one’s actions and to experience opportunities where one’s capacities can be exercised and expressed (Ryan and Deci, 2002). Thus, employees will likely feel more competent in an environment that provides opportunities to engage in challenging activities and that facilitates learning and skill development. Such an environment is, for example, given when the job is high in task identity and variety, according to Gagné and Panaccio (2014). Task identity may result in an increased feeling of mastery of one’s environment, while task and skill variety likely lead to the development of a broader skill set. A satisfied need for competence was, in turn, found to be associated with intrinsic work motivation, job satisfaction, subjective performance, and affect (van den Broeck et al., 2010; Meyer et al., 2012; Vandercammen et al., 2014). As a conclusion, we assumed that the positive effects of task variety, skill variety, and task identity on employees could be at least partly explained by the fact that these characteristics increased the experienced satisfaction of the need for competence. Thus, we assumed the following hypothesis.

*Hypothesis 2*: The effect of task rotation on expected (a) job satisfaction, (b) intrinsic work motivation, (c) subjective performance, (d) positive affect, and (e) negative affect is mediated by the parallel mediators perceived task variety, skill variety, and task identity, and the serial mediator expected satisfaction of the need for competence.

## Individual differences in work design effects

When implementing a work design intervention, such as task rotation, the goal is often that all employees benefit equally from the intervention to keep costs and effort to a minimum. However, past research has shown that the effects of work design differed depending on the employees’ personalities and other individual differences (e.g., Parker and Sprigg, 1999; Berdicchia et al., 2016). Thus, it is likely that there are also individual differences in the anticipated effects of task rotation.

An explanation for why an employee’s personality could influence the effect of task rotation on expected employee responses is provided by person-job fit theory (Edwards, 1991; Kristof-Brown et al., 2005). Person-job fit is defined as the match between an individual’s characteristics and those of the job or tasks that are performed in a job (Lu et al., 2014). A more specific form of person-job fit is needs-supplies fit, which occurs when an individual’s needs or preferences (e.g., a need to feel related to others) are satisfied by the characteristics of a job (e.g., a great degree of teamwork; Kristof, 1996). In their meta-analysis, Kristof-Brown et al. (2005) found that a high needs-supplies fit is associated with, for example, greater job satisfaction and performance, and lower strain.

As explained above, we have assumed that a job with task rotation provides the supplies of task variety, skill variety, and task identity. The Big Five personality factor openness to experience (McCrae, 1993) matches especially the supplies task variety and skill variety because individuals high on this factor are characterized by a preference for variety and novelty, an aversion to routines, a need for change, and an open mind. McCrae and Costa (1996) state that personality factors are relatively stable, endogenous basic tendencies that shape an individual’s thoughts, feelings, and behaviors. They claim that open individuals have an active motivation to explore and discover the unfamiliar and have more flexible attitudes when confronted with ambiguity or dissonance (McCrae, 1994; McCrae and Costa, 1997). A job with task rotation might provide an environment where open individuals can realize their personality—and consequently experience a great fit between personal needs and job supplies—because it offers a wide range of work activities that are not necessarily familiar in advance. De Jong et al. (2001) found, for example, that the relationship between skill variety and job satisfaction was stronger for individuals with higher openness to experience. Additionally, in a study by LePine et al. (2000), openness to experience was a relevant trait in changing task contexts. They found that individuals with higher openness performed significantly better at a decision-making task after there was an unforeseen change in the rules compared to individuals low in openness.

In conclusion, we expected the perceived task variety and skill variety to have stronger effects on the anticipated employee-related outcomes for individuals high in openness to experience. We did not expect this moderating effect for perceived task identity as there was no indication that it would provide a supply for an individual high in openness to experience.

*Hypothesis 3*: The effect of perceived task variety on (a) job satisfaction, (b) intrinsic work motivation, (c) subjective performance, (d) positive affect, and (e) negative affect and the effect of perceived skill variety on (f) job satisfaction, (g) intrinsic work motivation, (h) subjective performance, (i) positive affect, and (j) negative affect is moderated by openness to experience. For individuals with higher openness to experience, the effect will be stronger.

## Materials and methods

We tested our hypotheses in two consecutive studies. We used Study 1 to investigate the main effects and mediators. In Study 2, we added openness to experience as a potential moderator of the effects of task rotation on perceived work characteristics. The procedure for both studies was identical unless otherwise stated. The original contributions presented in the study are publicly available. This data can be found here: https://osf.io/XVKUA/.

### Participants

We recruited participants via personal contacts, social media, and in Study 1 via SurveyCircle (a survey sharing platform) as well. Participants in Study 1 could take part in a raffle for two gift certificates of 20 euros each. The incentive for participants in Study 2 was that one euro per participant was donated to animal rescue organizations. Before being able to participate in the studies, the participants were informed about the content and duration of the study, that their participation was completely voluntary and that they could terminate their participation at any time, and that their answers would be anonymous. Participants could only continue with the actual studies after they had read this information and had given their consent. We did not obtain written consent because data were analyzed anonymously. Ethical approval for both studies was obtained from the university’s ethics committee (IDs 2017-231 and 2019-189). There was a time lag of 1 year and 4 months between the data collections. An *a priori* power analysis revealed that we needed a minimum of 128 participants to find significant medium-sized (*d* = 0.5) main effects with a power of 0.80 and a significance level alpha of 0.05.

Although we described a concrete workplace in our vignettes (described in more detail in the “Procedure” Section), our focus was on the investigation of expected effects of task rotation on employee-related outcomes. Thus, the concrete work setting was irrelevant for our research. We therefore decided to recruit a heterogenous sample so that we were able to draw more generalized conclusions about potential effects of task rotation. The description of a concrete workplace served the purpose of eliciting more similar imaginations within an experimental condition than, for instance, when describing a generic task rotation situation. Discussions with six people from varying professional backgrounds before conducting the study ensured that participants could understand and imagine the described work situations.

A total of 177 participants in Study 1 and 199 participants in Study 2 completed the study. We excluded 42 participants in Study 1 and 40 participants in Study 2 because they either failed the manipulation check (“In the course of a working day, you conduct the same task the whole time. True or false?”) or checked the wrong box for a control question (“Please check the box that says ‘strongly agree”’). Therefore, our final sample consisted of 135 participants (*n* = 60 in the experimental condition, *n* = 75 in the control condition) in Study 1 and 159 participants (*n* = 77 in the experimental condition, *n* = 82 in the control condition) in Study 2. All participants were working a minimum of 17.5 h per week. In Study 1, they had a mean age of 32.36 years (*SD* = 11.46; *Min* = 19; *Max* = 62), 84 were female and 51 were male. In Study 2, the mean age was 41.38 years (*SD* = 12.35; *Min* = 19; *Max* = 67), 70 were female, 86 male, and three participants did not indicate their gender. Participants were asked to indicate their job title and, where possible, we assigned the corresponding code from the international standard classification of occupations (ISCO; International Labour Organization., 2016; see Table 1).

**TABLE 1:** Occupations of participants.

Occupation	Participants in Study 1	Participants in Study 2
Managers	6.7%	10.1%
Professionals	52.6%	56.6%
Technicians and associate professionals	7.4%	14.5%
Clerical support workers	9.6%	3.1%
Services and sales workers	5.2%	0.6%
Craft and related trades workers	2.2%	2.5%
Elementary occupations	0.7%	–
Plant and machine operators and assemblers	–	0.6%
Missing, or ISCO code could not be assigned	15.6%	11.9%

### Procedure

The study was administered online, and the manipulation consisted in a vignette that described an assembly workplace where the employee either rotated (experimental condition) or did not rotate (control condition) between work tasks. A vignette is a description of a fictional scenario, where certain characteristics can be manipulated and therefore experimentally investigated (Aguinis and Bradley, 2014). Compared to laboratory experiments and surveys in the field, experimental vignette studies have the major advantage that they enhance both the internal and external validity (Atzmüller and Steiner, 2010; Aguinis and Bradley, 2014): Internal validity is increased due to the experimental setting of the vignette, in which only the independent variables are manipulated and other environmental influences are controlled, while external validity is greater than in laboratory experiments because the vignettes describe scenarios that can happen in real life (Aguinis and Bradley, 2014). The experimental setting was necessary to investigate the causal effects of task rotation. That means, the results allow conclusions about the direction of the effect, and the possibility of confounding variables is controlled.

Experimental vignette studies are widely used in a range of research areas in applied psychology, including organizational justice (e.g., Bradley, 2006; Ötting and Maier, 2018), interpersonal relationships at work (e.g., Urbach et al., 2016; Thomas and Meglich, 2019), leadership (e.g., Steffens et al., 2018; Steinmann et al., 2020), or corporate social responsibility (e.g., Paruzel et al., 2020b). One might argue that vignettes are better suited for the investigation of hypothetical leaders, teams, or organizations, but there are also vignette studies in the field of work design research. van den Tooren and de Jonge (2010) examined, for example, hypothetical cognitively, emotionally, and physically demanding job situations and analyzed how relevant the matching job resources were perceived to be for regulating the demands. Zacher et al. (2017) created vignettes where certain work characteristics had varying degrees of intensity and examined how attractive these jobs were to the participants. Furthermore, there are several studies that examined the mediators and outcomes of the present study with the help of vignettes, more specifically work characteristics (Zacher et al., 2017), satisfaction of basic needs at work (Parmar et al., 2019), job satisfaction (Emery et al., 2019), intrinsic work motivation (Jacobsen and Jensen, 2017), positive and negative affect (Hohmann and Walter, 2019), and intended job performance (Drescher and Garbers, 2016).

The study had a two-factorial between-subjects design. Thus, at the beginning of the study, the participants were randomly assigned to one of two conditions. In Study 2, before the participants saw the vignette, they were asked to fill out the openness to experience questionnaire. In both studies, participants were then instructed to read the vignette thoroughly and imagine being the employee at the described workplace as best as they could. The vignettes in both experimental conditions described a workplace for a production mechanic responsible for the assembly of motors. The described work was supported by a digital assistance system that gave illustrated instructions for the necessary work steps. In the experimental condition, the vignette stated that the assistance system was also responsible for indicating a rotation between four tasks (cut material, assemble parts, check voltage, and analyze and fix errors), which occurred every 2 h. In the control condition, the vignette text described that the assistance system was responsible for indicating an appropriate time for a break. Participants in the control condition were also told that they performed the same task throughout the whole day and that their colleagues were responsible for the other three tasks. The task in the control condition was one of the four tasks described above, and participants in the control condition were randomly assigned to the respective vignettes.

The described workplace is based on a workplace that has been developed as part of a technological research project (cf. Oestreich et al., 2019). The four described tasks are supposed to reflect an assembly cycle: Cutting material happens during the preparation, assembling parts is the actual production, checking voltage is the control phase, and analyzing and fixing errors happens during postprocessing. Except for cutting materials, all tasks exist in the actual assistance system. However, they are only short simulations of about 2 min per task. Additionally, the system did not have task rotation implemented yet, which was the focus of this study. We therefore decided against conducting a laboratory experiment using the actual assistance system. Thus, we created the vignettes and discussed them with six people from varying professional backgrounds to ensure that they understood the scenario.

To increase immersion, we enriched the vignette text with a photo of an employee at the assistance system, as recommended by Aguinis and Bradley (2014). The vignette texts and photo can be found in the online Supplementary material (S1 Vignette in Supplementary material). To ensure that participants had read the vignettes thoroughly, they were then asked to answer multiple choice questions about the text. These were presented on the same page so that participants could reread the text. After reading the vignette, participants were asked to rate perceived work design characteristics, expected satisfaction of the need for competence, and several expected outcomes from the employee’s perspective.

### Measures

Except for openness to experience, all measures were prefaced by the instruction that all following questions referred to the situation that the participants were asked to imagine. They should answer all questions from this perspective, as if they were currently working in this workplace. Openness to experience was not prefaced by this instruction because participants should respond to the questions from their own perspective. It was also presented before showing the vignettes.

#### Openness to experience

We used the respective scale of the Big Five Inventory (John et al., 1991; German version: Rammstedt and John, 2005) to measure openness to experience. The scale consisted of 10 items assessed on a five-point Likert scale ranging from 1 (*does not apply at all*) to 5 (*applies very much*). A sample item is “I am someone who is original, comes up with new ideas” (Cronbach’s α = 0.79).

#### Work design characteristics

We assessed perceived task variety, skill variety, and task identity with the respective scales of the German version of the Work Design Questionnaire (Morgeson and Humphrey, 2006; Stegmann et al., 2010). All scales consisted of four items and were measured with a five-point Likert scale ranging from 1 (*strongly disagree*) to 5 (*strongly agree*). A sample item for task variety is “The job involves a variety of tasks,” (α = 0.95 in Study 1, 0.92 in Study 2); a sample item for skill variety is “The job requires the use of a number of skills,” (α = 0.94/0.89); and an example for task identity is “The job provides me the chance to completely finish the pieces of work I begin” (α = 0.85/0.84).

#### Satisfaction of the need for competence

We used the German translation of the Work-Related Basic Need Satisfaction Scale (van den Broeck et al., 2010; Martinek, 2014) to assess how much the participants expected their need for competence to be satisfied in the described workplace. The subscale consisted of six items and was measured with a five-point Likert scale ranging from 1 (*strongly disagree*) to 5 (*strongly agree*). A sample item is “I feel competent at my job” (α = 0.78/0.71).

#### Job satisfaction

We measured the overall job satisfaction with a single-item measure from Neuberger and Allerbeck (1978) that we adapted for this study. The adapted item is “When you think of everything that is important for your work (e.g., the work itself, working conditions), how satisfied would you be with your work as production mechanic overall?” Participants’ ratings were based on a seven-point Kunin scale (Kunin, 1955). According to a meta-analysis by Wanous et al. (1997), single-item measures of job satisfaction are highly correlated with scale measures of job satisfaction (*r* = 0.63).

#### Intrinsic work motivation

We used the subscale *intrinsic motivation* from the German version of the Multidimensional Work Motivation Scale to assess expected intrinsic work motivation (Gagné et al., 2015). It consists of three items and the ratings are based on a seven-point Likert scale from 1 (*strongly disagree*) to 7 (*strongly agree*). We adapted the stem of the scale as follows “Why would you put effort into the job as production mechanic?x” A sample item is “Because I have fun doing my job” (α = 0.95 in both studies).

#### Subjective performance

We measured the expected subjective performance in the described workplace with the self-constructed item “On a scale from 1 to 10, how high do you estimate your performance would be in the described job as a production mechanic based on your maximum performance capacity?” A 1 indicated low performance, a 10 referred to high performance. We indicated the maximum performance capacity as a reference value to ensure that ratings were comparable because it is less prone to intraindividual variations than typical performance (Fisher, 2008).

#### Affect

We measured the expected emotions and sentiments in the described workplace with the German adaptation of the Positive and Negative Affect Schedule (Watson et al., 1988; Krohne et al., 1996). The instrument consists of two scales with 10 adjectives each, and participants rated on a Likert scale from 1 (*not at all*) to 5 (*completely*) the extent to which each adjective would apply to them in their job as production mechanic. A sample item is “active” for the positive affect scale (α = 0.93/0.90) and “upset” for the negative affect scale (α = 0.89/0.86).

### Statistical analyses

To test our hypotheses, we conducted regression analyses using the PROCESS macro (version 3.0) for SPSS (version 23) by Hayes (2018). We conducted separate mediation analyses for each outcome. For each analysis, PROCESS generated 95% percentile bootstrap confidence intervals (thus, the significance level alpha was set to 0.05) using 5,000 bootstrap samples. To test for a moderated mediation, as assumed in Hypothesis 3, we investigated the index of moderated mediation. The index was developed by Hayes (2015) and indicates whether an indirect effect is dependent on a moderator. One can assume a moderated mediation when the confidence interval of this index does not include zero.

## Results

The zero-order correlations of study variables are depicted in Table 2. There were mostly significant correlations in the expected direction between mediators and outcome variables. The experimental manipulation (task rotation vs. no task rotation) was significantly related to perceived work characteristics, expected job satisfaction, expected intrinsic work motivation (only in Study 1), expected subjective performance (only in Study 2), and expected positive affect. There were no significant associations with the anticipated satisfaction of the need for competence and negative affect. To test for divergent validity, we closely investigated all correlation coefficients between two constructs with a value of 0.5 or higher. We calculated factor analyses with Promax rotation, and compared the variance extracted between both components within an analysis with the factor correlation squared. If the variance extracted between both components was higher, divergent validity was established. We did not perform this analysis with the constructs job satisfaction and subjective performance, as these were one-item measures, and a factor analysis could not be performed. The results showed divergent validity for all constructs (for more detailed results see S2 Table in the Supplementary material). The descriptive statistics and standardized mean differences of the experimental conditions are shown in Table 3. Participants in the task rotation condition gave higher ratings for all variables except expected negative affect, which was predicted. The effect sizes ranged from *d* = 0.26 to 1.53 in Study 1, which indicate medium to large effects (Bosco et al., 2015). In Study 2, effect sizes were mostly lower, especially regarding the expected satisfaction of the need for competence (*d* = −0.07 vs. 0.27) and negative affect (*d* = −0.02 vs. −0.26).

**TABLE 2:** Zero-order correlations of study variables.

Measure	1	2	3	4	5	6	7	8	9	10	11
1. Task rotation (0 = no rotation; 1 = rotation)	−	0.04	0.49***	0.27**	0.59***	–0.04	0.18*	0.13	0.17*	0.17*	–0.01
2. Openness to experience	−	−	0.03	0.09	0.15	0.17*	0.03	0.02	0.06	0.03	0.08
3. Perceived task variety	0.61**	−	−	0.70***	0.31***	0.13	0.35***	0.28***	0.33***	0.39***	–0.06
4. Perceived skill variety	0.44**	−	0.70**	−	0.27**	0.26**	0.42***	0.34***	0.35***	0.53***	−0.24**
5. Perceived task identity	0.50**	−	0.48**	0.49**	−	–0.10	0.26**	0.09	0.29***	0.26**	–0.14
6. Expected satisfaction of need for competence	0.13	−	0.20*	0.35**	0.33**	−	0.28***	0.14	0.21**	0.26**	−0.25**
7. Expected job satisfaction	0.19*	−	0.47**	0.61**	0.37**	0.36**	−	0.37***	0.53***	0.69***	−0.40***
8. Expected intrinsic work motivation	0.21*	−	0.41**	0.51**	0.36**	0.35**	0.53**	−	0.25**	0.46***	–0.14
9. Expected subjective performance	0.16	−	0.34**	0.50**	0.30**	0.34**	0.58**	0.48**	−	0.50***	−0.29***
10. Expected positive affect	0.18*	−	0.46**	0.61**	0.42**	0.41**	0.75**	0.58**	0.50**	−	−0.41***
11. Expected negative affect	–0.13	−	–0.10	−0.19*	–0.16	−0.44**	−0.25**	−0.32**	−0.26**	−0.22**	−

Correlations of Study 1 (*N* = 135) are presented below the diagonal, correlations of Study 2 (*N* = 159) are presented above the diagonal.

*p < 0.05; **p < 0.01; ***p < 0.001.

**TABLE 3:** Means and standard deviations of study variables, and standardized mean differences between experimental conditions.

	Study 1					Study 2				
		
	Task rotation		No task rotation			Task rotation		No task rotation		
						
Measure	*M*	*SD*	*M*	*SD*	*d*	*M*	*SD*	*M*	*SD*	*d*
Openness to experience	–	–	–	–	–	3.74	0.62	3.70	0.54	0.07
Perceived task variety	2.89	1.12	1.48	0.72	1.53	2.30	0.92	1.36	0.76	1.12
Perceived skill variety	3.78	1.22	2.47	1.06	1.16	2.47	0.98	1.94	0.94	0.55
Perceived task identity	3.16	1.04	2.12	1.08	0.98	3.82	1.14	2.23	1.07	1.44
Expected satisfaction of need for competence	3.69	0.76	3.49	0.71	0.27	3.40	0.66	3.45	0.75	−0.07
Expected job satisfaction	3.65	1.45	3.04	1.61	0.40	3.23	1.52	2.74	1.17	0.36
Expected intrinsic work motivation	4.04	1.82	3.25	1.80	0.44	3.89	1.93	3.37	1.93	0.27
Expected subjective performance	5.75	2.08	5.00	2.62	0.31	5.31	2.23	4.59	2.00	0.34
Expected positive affect	2.41	0.82	2.11	0.80	0.37	2.24	0.79	1.99	0.63	0.35
Expected negative affect	1.50	0.66	1.67	0.65	−0.26	1.62	0.66	1.64	0.64	−0.02

Study 1: *n* = 60 in the task rotation condition, *n* = 75 in the no task rotation condition; Study 2: *n* = 77 in the task rotation condition, *n* = 82 in the no task rotation condition.

*d*, Standardized mean difference Cohen’s *d*.

### Hypothesis testing

An overview of all hypotheses and whether they were supported or rejected in our studies can be found in Table 4. The regression coefficients, standard errors, and model summaries can be found in Table 5 (Study 1) and Table 6 (Study 2). In Hypothesis 1, we predicted that task rotation had a positive effect on various expected employee-related outcomes. In Hypothesis 2, we stated that this main effect would be mediated by the parallel mediators perceived task variety, skill variety, and task identity, and the serial mediator expected satisfaction of the need for competence. To examine Hypothesis 1, we investigated the total effects in Table 7 (Study 1) and Table 8 (Study 2). In Study 1, we found that task rotation had a significant positive effect on expected job satisfaction (*b* = 0.61, *p* = 0.02), intrinsic work motivation (*b* = 0.79, *p* = 0.01), and positive affect (*b* = 0.30, *p* = 0.03). The effect of task rotation on expected subjective performance (*b* = 0.75, *p* = 0.07) and negative affect (*b* = −0.17, *p* = 0.13) was not significant. In Study 2, we could replicate these findings regarding expected job satisfaction (*b* = 0.49, *p* = 0.02), positive affect (*b* = 0.25, *p* = 0.03), and negative affect (*b* = −0.01, *p* = 0.89), but the effect of task rotation on expected intrinsic work motivation was no longer significant (*b* = 0.52, *p* = 0.09), and the previously insignificant effect on expected subjective performance became significant (*b* = 0.73, *p* = 0.03). In conclusion, we found support for Hypothesis 1a and 1d, and partial support for Hypothesis 1b and 1c. In both studies, we could not find supporting evidence for Hypothesis 1e.

**TABLE 4:** Overview of supported and rejected hypotheses.

Hypothesis	Study 1	Study 2
H1a: Task rotation → expected job satisfaction	✓	✓
H1b: Task rotation → expected intrinsic work motivation	✓	ns
H1c: Task rotation → expected subjective performance	ns	✓
H1d: Task rotation → expected positive affect	✓	✓
H1e: Task rotation → expected negative affect	ns	ns
H2a: Perceived task variety, skill variety, and task identity, and the expected satisfaction of the need for competence mediate task rotation → expected job satisfaction	✓	✓
H2b: Perceived task variety, skill variety, and task identity, and the expected satisfaction of the need for competence mediate task rotation → expected intrinsic work motivation	✓	ns
H2c: Perceived task variety, skill variety, and task identity, and the expected satisfaction of the need for competence mediate task rotation → expected subjective performance	✓	✓
H2d: Perceived task variety, skill variety, and task identity, and the expected satisfaction of the need for competence mediate task rotation → expected positive affect	✓	✓
H2e: Perceived task variety, skill variety, and task identity, and the expected satisfaction of the need for competence mediate task rotation → expected negative affect	ns	ns
H3a: Perceived task variety → expected job satisfaction is moderated by openness to experience	—	ns
H3b: Perceived task variety → expected intrinsic work motivation is moderated by openness to experience	—	ns
H3c: Perceived task variety → expected subjective performance is moderated by openness to experience	—	ns
H3d: Perceived task variety → expected positive affect is moderated by openness to experience	—	ns
H3e: Perceived task variety → expected negative affect is moderated by openness to experience	—	ns
H3f: Perceived skill variety → expected job satisfaction is moderated by openness to experience	—	ns
H3g: Perceived skill variety → expected intrinsic work motivation is moderated by openness to experience	—	ns
H3h: Perceived skill variety → expected subjective performance is moderated by openness to experience	—	ns
H3i: Perceived skill variety → expected positive affect is moderated by openness to experience	—	ns
H3j: Perceived skill variety → expected negative affect is moderated by openness to experience	—	ns

Checkmark symbol indicates supported hypotheses, ns indicate rejected hypotheses.

**TABLE 5:** Regression coefficients, standard errors, and model summary for all outcomes in Study 1.

	(1) DV: Expected job satisfaction		(2) DV: Expected intrinsic work motivation		(3) DV: Expected subjective performance		(4) DV: Expected positive affect		(5) DV: Expected negative affect	
					
Predictor	*b* (*SE*)	95% CI	*b* (*SE*)	95% CI	*b* (*SE*)	95% CI	*b* (*SE*)	95% CI	*b* (*SE*)	95% CI
Constant	−0.07 (0.53)	[−1.13, 0.99]	−0.17 (0.69)	[−1.53, 1.19]	0.73 (0.91)	[−1.08, 2.54]	0.25 (0.27)	[−0.29, 0.79]	2.99 (0.26)	[2.47, 3.51]
Task rotation	−0.61 (0.28)	[−1.16, −0.06]	−0.39 (0.36)	[−1.10, 0.32]	−0.58 (0.48)	[−1.52, 0.36]	−0.36 (0.14)	[−0.65, −0.08]	−0.15 (0.14)	[−0.42, 0.12]
Perceived task variety	0.26 (0.14)	[−0.02, 0.54]	0.23 (0.19)	[−0.13, 0.60]	0.11 (0.25)	[−0.38, 0.60]	0.12 (0.07)	[−0.03, 0.26]	0.04 (0.07)	[−0.10, 0.18]
Perceived skill variety	0.60 (0.13)	[0.34, 0.86]	0.51 (0.17)	[0.17, 0.85]	0.85 (0.23)	[0.40, 1.30]	0.30 (0.07)	[0.17, 0.43]	−0.03 (0.07)	[−0.16, 0.10]
Perceived task identity	0.13 (0.10)	[−0.08, 0.33]	0.18 (0.13)	[−0.08, 0.44]	0.14 (0.17)	[−0.21, 0.48]	0.11 (0.05)	[0.01, 0.22]	0.02 (0.05)	[−0.08, 0.12]
Expected satisfaction of need for competence	0.32 (0.16)	[0.02, 0.63]	0.44 (0.20)	[0.05, 0.84]	0.56 (0.27)	[0.03, 1.09]	0.22 (0.08)	[0.06, 0.38]	−0.39 (0.08)	[−0.54, −0.23]
	*R*^2^ = 0.42		*R*^2^ = 0.31		*R*^2^ = 0.29		*R*^2^ = 0.45		*R*^2^ = 0.20	
	*F*(5,129) = 18.79, *p* < 0.001		*F*(5,129) = 11.52, *p* < 0.001		*F*(5,129) = 10.33, *p* < 0.001		*F*(5,129) = 21.29, *p* < 0.001		*F*(5,129) = 6.62, *p* < 0.001	

*N* = 135. Unstandardized regression coefficients are reported.

DV, dependent variable; CI, confidence interval.

**TABLE 6:** Regression coefficients, standard errors, and model summary for all outcomes in Study 2.

	(1) DV: Expected job satisfaction		(2) DV: Expected intrinsic work motivation		(3) DV: Expected subjective performance		(4) DV: Expected positive affect		(5) DV: Expected negative affect	
					
Predictor	*b* (*SE*)	95% CI	*b* (*SE*)	95% CI	*b* (*SE*)	95% CI	*b* (*SE*)	95% CI	*b* (*SE*)	95% CI
Constant	−0.10 (0.55)	[−1.18, 0.99]	1.71 (0.84)	[0.04, 3.37]	0.64 (0.89)	[−1.12, 2.39]	0.56 (0.28)	[0.01, 1.10]	2.70 (0.28)	[2.15, 3.25]
Task rotation	−0.17 (0.26)	[−0.69, 0.35]	0.17 (0.40)	[−0.63, 0.97]	−0.48 (0.43)	[−1.33, 0.36]	−0.09 (0.13)	[−0.36, 0.17]	0.10 (0.13)	[−0.16, 0.36]
Perceived task variety	0.17 (0.16)	[−0.14, 0.48]	0.16 (0.24)	[−0.32, 0.63]	0.43 (0.25)	[−0.07, 0.93]	0.04 (0.08)	[−0.12, 0.20]	0.12 (0.08)	[−0.03, 0.28]
Perceived skill variety	0.32 (0.14)	[0.04, 0.61]	0.52 (0.22)	[0.08, 0.95]	0.26 (0.23)	[−0.20, 0.72]	0.30 (0.07)	[0.16, 0.44]	−0.19 (0.07)	[−0.33, −0.04]
Perceived task identity	0.22 (0.09)	[0.05, 0.40]	−0.04 (0.14)	[−0.31, 0.23]	0.44 (0.15)	[0.15, 0.72]	0.10 (0.05)	[0.01, 0.19]	−0.09 (0.05)	[−0.18, −0.00]
Expected satisfaction of need for competence	0.43 (0.14)	[0.15, 0.71]	0.16 (0.22)	[−0.28, 0.59]	0.55 (0.23)	[0.09, 1.00]	0.17 (0.07)	[0.02, 0.31]	−0.19 (0.07)	[−0.34, −0.05]
	*R*^2^ = 0.25		*R*^2^ = 0.12		*R*^2^ = 0.20		*R*^2^ = 0.32		*R*^2^ = 0.14	
	*F*(5,153) = 10.09, *p* < 0.001		*F*(5,153) = 4.28, *p* = 0.001		*F*(5,129) = 7.76, *p* < 0.001		*F*(5,129) = 14.14, *p* < 0.001		*F*(5,129) = 4.82, *p* < 0.001	

*N* = 159. Unstandardized regression coefficients are reported.

DV, dependent variable; CI, confidence interval.

**TABLE 7:** Total and indirect effects in Study 1.

	(1) DV: Expected job satisfaction		(2) DV: Expected intrinsic work motivation		(3) DV: Expected subjective performance		(4) DV: Expected positive affect		(5) DV: Expected negative affect	
					
Effect	*b* (*SE*)	95% CI	*b* (*SE*)	95% CI	*b* (*SE*)	95% CI	*b* (*SE*)	95% CI	*b* (*SE*)	95% CI
Total effect	0.61 (0.27)	[0.08, 1.14]	0.79 (0.31)	[0.17, 1.41]	0.75 (0.41)	[−0.07, 1.57]	0.30 (0.14)	[0.02, 0.58]	−0.17 (0.11)	[−0.40, 0.05]
Total indirect effect	1.22 (0.24)	[0.76, 1.69]	1.18 (0.33)	[0.61, 1.88]	1.33 (0.37)	[0.60, 2.07]	0.67 (0.15)	[0.40, 0.99]	−0.03 (0.13)	[−0.30, 0.20]
TR → perceived task variety → DV	0.37 (0.19)	[0.00, 0.77]	0.33 (0.32)	[−0.26, 1.00]	0.16 (0.32)	[−0.48, 0.80]	0.16 (0.11)	[−0.04, 0.41]	0.06 (0.10)	[−0.16, 0.24]
TR → perceived skill variety → DV	0.63 (0.18)	[0.29, 1.02]	0.53 (0.21)	[0.16, 0.96]	0.88 (0.28)	[0.37, 1.48]	0.31 (0.10)	[0.13, 0.53]	−0.03 (0.07)	[−0.16, 0.10]
TR → perceived task identity → DV	0.16 (0.13)	[−0.10, 0.42]	0.24 (0.21)	[−0.16, 0.67]	0.18 (0.21)	[−0.25, 0.60]	0.15 (0.07)	[0.02, 0.29]	0.02 (0.06)	[−0.11, 0.14]
TR → expected need satisfaction → DV	−0.03 (0.06)	[−0.17, 0.08]	−0.05 (0.08)	[−0.23, 0.11]	−0.06 (0.11)	[−0.30, 0.14]	−0.02 (0.04)	[−0.10, 0.05]	0.04 (0.07)	[−0.07, 0.20]
TR → perceived task variety → expected need satisfaction → DV	−0.04 (0.05)	[−0.15, 0.04]	−0.05 (0.06)	[−0.18, 0.07]	−0.06 (0.08)	[−0.24, 0.08]	−0.02 (0.03)	[−0.09, 0.03]	0.04 (0.05)	[−0.05, 0.13]
TR → perceived skill variety → expected need satisfaction → DV	0.07 (0.05)	[−0.01, 0.19]	0.10 (0.06)	[−0.01, 0.24]	0.12 (0.08)	[0.01, 0.30]	0.05 (0.03)	[0.01, 0.11]	−0.09 (0.04)	[−0.17, −0.02]
TR → perceived task identity → expected need satisfaction → DV	0.06 (0.04)	[−0.01, 0.16]	0.08 (0.06)	[−0.01, 0.21]	0.10 (0.07)	[0.00, 0.26]	0.04 (0.02)	[0.01, 0.09]	−0.07 (0.04)	[−0.17, −0.01]

*N* = 135. Unstandardized regression coefficients are reported.

DV, dependent variable; CI, confidence intervals; TR, task rotation.

**TABLE 8:** Total and indirect effects in Study 2.

	(1) DV: Expected job satisfaction		(2) DV: Expected intrinsic work motivation		(3) DV: Expected subjective performance		(4) DV: Expected positive affect		(5) DV: Expected negative affect	

Effect	*b* (*SE*)	95% CI	*b* (*SE*)	95% CI	*b* (*SE*)	95% CI	*b* (*SE*)	95% CI	*b* (*SE*)	95% CI
Total effect	0.49 (0.21)	[0.07, 0.91]	0.52 (0.31)	[−0.09, 1.12]	0.73 (0.34)	[0.06, 1.39]	0.25 (0.11)	[0.02, 0.47]	−0.01 (0.10)	[−0.22, 0.19]
Total indirect effect	0.66 (0.23)	[0.24, 1.14]	0.35 (0.30)	[−0.30, 0.66]	1.21 (0.32)	[0.60, 1.89]	0.34 (0.14)	[0.10, 0.63]	−0.11 (0.11)	[−0.33, 0.09]
TR → perceived task variety → DV	0.16 (0.20)	[−0.16, 0.64]	0.15 (0.24)	[−0.30, 0.66]	0.40 (0.27)	[−0.09, 0.99]	0.04 (0.10)	[−0.13, 0.28]	0.12 (0.08)	[−0.06, 0.27]
TR → perceived skill variety → DV	0.17 (0.10)	[0.00, 0.39]	0.27 (0.15)	[0.04, 0.63]	0.14 (0.14)	[−0.13, 0.43]	0.16 (0.06)	[0.06, 0.29]	−0.10 (0.04)	[−0.20, −0.03]
TR → perceived task identity → DV	0.36 (0.14)	[0.09, 0.64]	−0.07 (0.23)	[−0.53, 0.36]	0.70 (0.24)	[0.23, 1.20]	0.16 (0.08)	[0.01, 0.31]	−0.14 (0.07)	[−0.29, −0.02]
TR → expected need satisfaction → DV	0.01 (0.07)	[−0.14, 0.14]	0.00 (0.04)	[−0.09, 0.09]	0.01 (0.09)	[−0.17, 0.20]	0.00 (0.03)	[−0.05, 0.06]	−0.00 (0.03)	[−0.07, 0.06]
TR → perceived task variety → expected need satisfaction → DV	−0.02 (0.04)	[−0.11, 0.06]	−0.01 (0.03)	[−0.06, 0.05]	−0.03 (0.06)	[−0.17, 0.07]	−0.01 (0.02)	[−0.05, 0.02]	0.01 (0.02)	[−0.03, 0.06]
TR → perceived skill variety → expected need satisfaction → DV	0.06 (0.04)	[0.01, 0.15]	0.02 (0.03)	[−0.04, 0.10]	0.07 (0.05)	[0.00, 0.20]	0.02 (0.02)	[0.00, 0.06]	−0.03 (0.02)	[−0.07, −0.00]
TR → perceived task identity → expected need satisfaction → DV	−0.06 (0.04)	[−0.17, 0.00]	−0.02 (0.04)	[−0.11, 0.05]	−0.08 (0.06)	[−0.22, 0.01]	−0.03 (0.02)	[−0.07, 0.00]	0.03 (0.02)	[−0.00, 0.08]

*N* = 159. Unstandardized regression coefficients are reported.

DV, dependent variable; CI, confidence intervals; TR, task rotation.

To investigate Hypothesis 2, we calculated and tested the total indirect effects (cf. Tables 7, 8). A significant indirect effect indicates mediation (Hayes, 2009). The confidence interval of the indirect effect did not include zero, which means that the effect was significant for expected job satisfaction, intrinsic work motivation (only in Study 1), subjective performance, and positive affect. Thus, we found support for Hypothesis 2a, 2c, and 2d, and partial support for Hypothesis 2b. In both studies, we could not find evidence for an indirect effect on expected negative affect. Therefore, we had to reject Hypothesis 2e. Upon closer inspection of the single indirect effects, one can see that the significant indirect effects mainly involved perceived skill variety or task identity, partly combined with the expected satisfaction of the need for competence. Hence, skill variety and task identity can be considered the strongest mediators.

Lastly, in Hypothesis 3, we assumed that openness to experience would moderate the effects of perceived task variety and skill variety on the outcomes. To investigate this hypothesis, we added the interactions between task variety and openness and between skill variety and openness to the existing model. The results showed that the addition of the interaction terms did not significantly increase the percentage of variance explained for anticipated job satisfaction [task variety: Δ*R*^2^ = 0.00, *F*(1,150) = 0.08, *p* = 0.77; skill variety: Δ*R*^2^ = 0.00, *F*(1,150) = 0.40, *p* = 0.53], intrinsic work motivation [task variety: Δ*R*^2^ = 0.01, *F*(1,150) = 1.23, *p* = 0.27; skill variety: Δ*R*^2^ = 0.00, *F*(1,150) = 0.24, *p* = 0.62], subjective performance [task variety: Δ*R*^2^ = 0.01, *F*(1,150) = 1.72, *p* = 0.19; skill variety: Δ*R*^2^ = 0.00, *F*(1,150) = 0.40, *p* = 0.53], positive affect [task variety: Δ*R*^2^ = 0.00, *F*(1,150) = 0.42, *p* = 0.52; skill variety: Δ*R*^2^ = 0.00, *F*(1,150) = 0.00, *p* = 0.97], or negative affect [task variety: Δ*R*^2^ = 0.01, *F*(1,150) = 1.25, *p* = 0.27; skill variety: Δ*R*^2^ = 0.00, *F*(1,150) = 0.01, *p* = 0.91]. Additionally, the indices of moderated mediation were non-significant for all indirect effects. Thus, we rejected Hypothesis 3 (for more detailed results see S3 Table and S4 Table in the Supplementary material).

As the samples in Study 1 and Study 2 differed significantly in terms of age and gender, we repeated all analyses with these variables as covariates. The analyses yielded comparable results. Thus, differences in the results of the two studies are not due to different sample compositions.

## Discussion

Our aim in the present research was to investigate the work design method task rotation as a technology feature of digital assistance systems in more detail. More specifically, we examined whether associations between task rotation and positive work attitudes, behavior, and well-being from previous studies were due to unique effects of task rotation and could also be expected when task rotation was implemented as a feature of technology, by which constructs these effects could be explained, and whether there were individual differences in the effects. To this end, we conducted two consecutive experimental vignette studies in which participants imagined working at a workplace with a digital assistance system that either prescribed a task rotation every 2 h (experimental condition) or not (control condition). Consistent with claims for more context-sensitive research (e.g., Johns, 2006; Morgeson et al., 2010), we investigated task rotation in the context of a digitally assisted workplace. That means, our goal was to investigate the effects of task rotation in this specific setting, and not compare a digitally assisted rotation with a rotation organized by a human.

We found that participants consistently anticipated positive effects of task rotation on job satisfaction and positive affect. In one study each, task rotation positively affected the expected intrinsic work motivation and subjective performance. In both studies, there was no effect of task rotation on anticipated negative affect. We further found that there were consistent indirect effects of task rotation, transmitted by perceived task variety, skill variety, and task identity in parallel, and expected satisfaction of the need for competence as a serial mediator, on expected job satisfaction, subjective performance, and positive affect. An indirect effect on expected intrinsic work motivation that we found in the first study could not be replicated in the second study. There were no indirect effects on expected negative affect in either of the studies. Lastly, we could not find evidence for individual differences in effects of perceived task and skill variety on anticipated positive employee responses due to the participants’ openness to experience.

As outlined above, we were not able to support all our assumptions consistently. An explanation is that there was much variation across participants in both conditions. For example, the total effect of task rotation on expected subjective performance was greater in Study 1 (*b* = 0.75) than in Study 2 (*b* = 0.73) but became significant only in Study 2 due to less variation in participants’ responses. We had deliberately chosen to use a sample with diverse professional backgrounds so that the results applied to a broader population. However, a more homogenous sample might have produced more consistent results. Furthermore, it is possible that the results for expected job satisfaction and affect were consistent across studies because these have an affective component and are therefore more immediate responses. By contrast, intrinsic work motivation as an attitudinal response and subjective performance as a behavioral response might be more distal because they are determined by affective evaluations (Ajzen, 1991). Consequently, it should be considered that the experimental vignette setting could have made it harder for participants to imagine their attitudinal and behavioral responses, as opposed to the more proximal affective responses.

Still, we were unable to support our assumption that task rotation leads to significantly less anticipated negative affect than no rotation. An explanation for this result could be that although positive work design or technology features can increase positive affect, the absence of these features does not necessarily increase negative affect because individuals do not know that the job could have more positive features (Warr et al., 1983; Watson, 1988). This issue becomes especially apparent in a between-subjects design, which was adopted in this study. While the work with task rotation could be perceived as pleasant, it is possible that the work depicted in the control condition was not perceived as unpleasant, but rather as neutral. This is supported by the fact that a non-rotating workplace is common for many people. In the CRANET survey of 2014/15 (Cranet., 2017), almost half of the European organizations reported that they had not adopted job rotation, which might be a proxy for the adoption of task rotation.

Another result that deserves special attention is that openness to experience did not moderate the effects of perceived task and skill variety on anticipated employee-related outcomes. As there was, however, much variation in participants’ responses, it is possible that there are other moderating variables that we did not investigate. Another explanation could be that, according to McCrae and Costa (1997), the majority of people are intermediate in openness. This knowledge, combined with the medium-sized means and small standard deviations of our sample (see Table 3), could explain that there might have been too little variance in openness to experience to detect an effect. Then again, it is also possible that the tasks described in the rotation condition did not affect people who are more open to experience because they were routine tasks, and not any unexpected activities.

### Theoretical implications

Although task rotation has been practiced and researched for a long time, it has not yet been investigated as a technology feature and its inner workings were still a ‘black box.’ In addressing these research gaps, we successfully combined several theories from work design that have not yet been investigated together.

First, we combined Parker et al.’s (2017a) framework of work design influences with the work design framework by Morgeson and Humphrey (2006, 2008), which is based on the JCM by Hackman and Oldham (1976). The former describes contextual influences, such as the human resources practice task rotation, on work design characteristics. The latter illustrates how work characteristics in turn lead to employee-related effects, such as attitudes, affect, or behavior. So far, empirical studies mainly had a focus on the association between work characteristics and employee outcomes, but excluded the contextual factors. This is also reflected in Morgeson and Humphrey (2006) meta-analysis on this subject. This is problematic because, in order to ensure good work design, and react to changes brought about by technological advancements or societal developments, it is also important to know what affects work design. As Parker et al. (2017a) stated, most theory and research about work design treats it as an independent variable, which does not allow any conclusions about the origins of work design. Thus, our manuscript provides an important step to shed light on at least one contextual factor of work design, meaning the human resource practice task rotation.

Second, we also combined self-determination theory (e.g., Deci and Ryan, 2014) with the work design framework, or its predecessor the JCM (Hackman and Oldham, 1976). This contributes to work design knowledge because mediators from the JCM were not entirely supported in meta-analytical observations (Humphrey et al., 2007). More specifically, the JCM states that the associations between skill variety and task identity (task variety not being part of the model at all) and employee-related outcomes are mediated by experienced meaningfulness. A full mediation could be found in Humphrey et al.’s (2007) meta-analysis only for the outcomes job satisfaction and internal work motivation. The relationships with subjective performance were only partially mediated, and other outcomes could not be investigated at all due to a lack of primary studies. Thus, Gagné and Panaccio (2014) had suggested to add mediators from self-determination theory. More specifically, the authors illustrate parallels between self-determination theory and the JCM. For example, they state that increasing task identity and variety at a job should contribute to an employee’s feeling of competence. As we found evidence for the serial mediator satisfaction of the need for competence, this is a first indication that self-determination theory actually does offer alternatives to the mediators of the JCM. To further investigate the combination of the JCM and self-determination theory, research on other work design methods, which might affect the satisfaction of the needs for relatedness or autonomy, would be necessary. These were not within the scope of this study, as the need for competence was the most fitting with regard to task rotation effects.

Lastly, our studies contribute knowledge to task rotation in specific. Since we adopted an experimental approach, we found evidence for unique, causal effects of task rotation on positive employee-related outcomes, such as the anticipated job satisfaction and positive affect. This adds to the existing knowledge on task rotation because previous studies were mainly correlational, and therefore could not exclude alternative explanations for the association between task rotation and another variable, such as confounding effects. A possible confounding effect could be created by other so-called high-performance work practices. Practices falling under this umbrella term aim at improving an organization by attracting or developing high-performing employees (e.g., Cappelli and Neumark, 2001). The fact that task rotation is often regarded as one high-performance work practice of many is an indication that companies adopting task rotation might also adopt related practices, like self-managed teams (i.e., teams that decide without a supervisor how to perform tasks or which tasks to perform). Therefore, correlational effects between task rotation and, for example, job satisfaction, might have been only due to one of the other practices. Based on previous, correlational studies, it was not clear whether positive relationships could really be traced back to the task rotation.

### Practical implications

The increasing adoption of technologies at work poses new challenges to occupational and organizational psychologists (Cascio and Montealegre, 2016; Landers and Marin, 2021). Our studies may show that the introduction of a digital assistance system does not determine effects on employees *per se*, but that these also depend on the concrete technology features, in this case the presence or absence of task rotation. The former case, that is a deterministic view of technology, is what Landers and Marin called “technology-as-causal” in their review on how technology is integrated in occupational and organizational psychology research. It would have meant to merely compare a workplace with a digital assistance system with a workplace without such a system. The problem with such an approach is that it is uncertain how well the results of the studies would generalize to any other digital assistance system. Thus, by focusing on a specific feature, in this case the existence or absence of task rotation, our results may generalize to other systems that provide this feature. Consequently, companies planning to implement a new technology should consider the concrete features and their resulting motivational effects already in the early stages of the technology design process.

In the sense of the sociotechnical systems approach (Cherns, 1987; Davis et al., 2014) and sociomateriality (Orlikowski and Scott, 2008; Landers and Marin, 2021), technology designers should work together with occupational psychologists to ensure that the technical system (i.e., the digital system and adjacent technologies) and the social system (consisting of the employees and the organization) are optimized in harmony with each other. Our results suggest that task rotation can be one way to improve jobs that run the risk of becoming more monotonous when technology is implemented. The fact that a digital assistance system gives so much guidance that training efforts can be reduced makes task rotation also a quite affordable work design technique. A further interesting aspect is that a study by Della Torre and Solari (2011) found that the combined investment in high-performance work practices, such as task rotation, and advanced technologies resulted in the greatest labor productivity and economic performance, as opposed to the sole investment in either technologies or work practices. The authors even found that companies with lower technological levels, a high degree of organizational practices could even be regarded as counter-productive, as it resulted in worse business results.

Regarding the concrete design of task rotation interventions, our studies also give indications. As we found that skill variety and task identity were the strongest mediators in our studies, companies should not simply focus on adding a range of different tasks to a rotation cycle. Instead, the tasks within a rotation should require a diverse set of skills and make up a complete work process. This is a new approach, as previous research was mainly concerned with the number of tasks (e.g., Jeon et al., 2016), but not their content or required skills. A practical approach to ensuring skill variety could be examining how the occupational information network database defines each skill^1^. For example, the requirement *operation monitoring* is defined as “watching gauges, dials, or other indicators to make sure a machine is working properly” (National Center for O*Net Development., 2022). Thus, it would not be effective to let employees rotate between several tasks that each requires them to observe some sort of performance indicator. Instead, they could rotate regularly between, for example, monitoring and troubleshooting (“determining causes of operating errors and deciding what to do about it”; National Center for O*Net Development., 2022). That way, the employees would be responsible for a greater part of the work process (because they do not have to rely on a specialist who helps with the troubleshooting), and the job would require different skills.

### Limitations and directions for future research

A common criticism of experimental research is a lack of external validity that compromises the generalizability of results (Scandura and Williams, 2000). It should therefore be noted that the results of the studies can only be interpreted in terms of prospective work design. As we investigated a hypothetical scenario in an experimental setting, effects of task rotation in complex real work settings might differ. One could expect differences between vignette and field study especially regarding the outcomes subjective performance and intrinsic work motivation. As we have outlined above, these are more distal responses compared to the affective reactions (cf. Ajzen, 1991). Thus, a vignette can probably not capture these outcomes as reliably. Yet, we had two important reasons to prefer an experimental vignette study to a field study. First, vignette studies offer the unique possibility to investigate scenarios that do not yet exist in the field. This aspect is highly relevant in current workplaces that are affected by fast-changing technologies. For technology designers, it is important to know about the expected consequences of certain technology features while the technologies are still being developed and not when they have already been implemented. Nevertheless, we encourage future researchers to replicate our studies in the field once digital assistance systems with task rotation have become more widely established in real work settings. Second, one aim of our research was to investigate whether there were unique effects of task rotation on the expected outcomes. In field studies, there are usually confounding environmental factors. As an example, it is possible that the departments adopting task rotation are newly founded so that the employees have new colleagues, which can also affect how they feel about their job. To further increase immersion, future research could build on our results and investigate mechanisms of task rotation in microworld simulations. Microworlds are virtual environments that participants interact with and that simulate situations that could happen in real work settings (Difonzo et al., 1998; Romme, 2004). As their development and programming can be resource-intensive, this method was not appropriate for a first assessment of task rotation as a technology feature.

A further limitation of our research is a potential common method bias, because we assessed most variables via self-report (Podsakoff et al., 2003). However, there can be no common method bias in the investigated main effects because we experimentally manipulated the independent variable. Regarding the expected mediator and moderator effects, our methodological approach of conducting a vignette study restricted us from using different sources of information. This is usually a recommended remedy against common method bias (Podsakoff et al., 2012), but is not feasible when all questions concern the participants’ perceptions in a fictional scenario. Conway and Lance (2010) even stated that self-report measures were appropriate when the targeted information involved perceptions, rather than objective data.

As a further direction for future research, we suggest that other individual differences should be examined as possible moderators. We focused on openness to experience because it is one of the basic personality factors (McCrae and Costa, 1996), but it is possible that the anticipated effects of task rotation on employee-related outcomes rather are affected by more work-related moderators, such as proactive personality. Following the reasoning of Zhang et al. (2019), one could expect that the effects are stronger when proactive personality is low, because less proactive employees are more dependent on the resources given by their job than more proactive employees, who can provide for their resources through their proactive behavior.

## Conclusion

The increasing adoption of advanced technologies that affect great parts of the work process could make some jobs more specialized and monotonous. In two studies, we attempted to show that task rotation could be a suitable technology feature to counteract potential negative effects. By increasing the perceived task variety, skill variety, and task identity, task rotation is expected to satisfy the need for competence, which particularly affects employees’ expected job satisfaction and positive affect.

## Data availability statement

The datasets presented in this study can be found in online repositories. The names of the repository/repositories and accession number(s) can be found below: https://osf.io/XVKUA/.

## Ethics statement

The studies involving human participants were reviewed and approved by Ethics Committee of Bielefeld University. Written informed consent for participation was not required for this study in accordance with the national legislation and the institutional requirements.

## Author contributions

LM, JL, and GM contributed to conception and design of the study. LM performed statistical analyses and wrote the manuscript. LM and GM contributed to manuscript revision, read, and approved the submitted version. All authors contributed to the article and approved the submitted version.

## Footnotes

1
https://www.onetonline.org/
