# Supplementary material for: New work situations call for familiar work design methods: Effects of task rotation and how they are mediated in a technology-supported workplace
Source: Front Psychol. 2022 Oct 12;13:935952. doi: 10.3389/fpsyg.2022.935952 (PMC9597497; doi:10.3389/fpsyg.2022.935952)
Supplement: Supplementary file 1 [file Data_Sheet_1.PDF]

Supplementary Material

1 Vignette. Vignette texts and photo

Introduction for Both Conditions

You work as a production mechanic in the engine assembly department of a well-known company, which manufactures various motorized children's toys, such as a driving toy car. You work with a digital assistance system, as depicted in the photo *(see Figure 1)*. The system supports you in your work, for example, by showing you the subsequent working steps in pictures.

Vignette Text for Experimental Condition

The system is also responsible for deciding when and at what frequency a task change is due. This means, after a certain time, the assistance system will tell you which task you should do next.

During your working hours, you always switch between the following tasks related to the manufacturing process of a child's toy: You cut the raw material for a toy into shape, or you assemble the smoothed parts according to the digital assistance system instructions to create a finished product. A further task is to check that the toy is working properly with a voltage tester, which is connected to the assistance system. The last possible task is to analyze and correct defects in non-functioning toys with the help of a manual in the digital assistance system.

You switch between tasks such that you have done each task exactly once each day and have thus gone through a complete work cycle.

Example Vignette Text for Control Condition

The system is also responsible for informing you when a break could be taken. That means, after a certain time, the assistance system tells you when a breakfast or lunch break is possible in the workflow.

During your working hours, you always work on the following task in the manufacturing process of a child's toy: You analyze and correct defects in non-functioning toys with the help of a manual in the digital assistance system *(the three other vignette texts in the control condition contained one of the other tasks at the workplace)*. There are also other tasks in the production process, but these are carried out by your work colleagues. Some colleagues cut the raw material for a toy into shape. Others assemble the smoothed parts according to the digital assistance system instructions to create a finished product, while still others check that the toy is working properly with a voltage tester, which is connected to the assistance system. You are specialized in analyzing and correcting defects in the toys. You perform this task every day and do not switch between different tasks.

Figure 1

Photo depicted as part of the experimental vignette

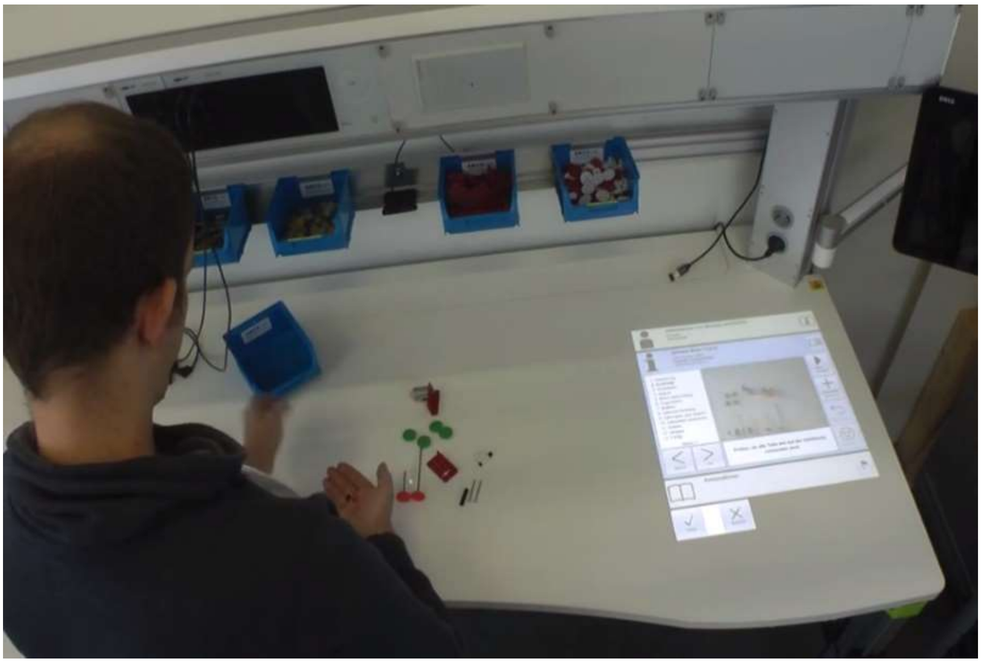

2 Table. Comparison of variance extracted between components and factor correlation squared

	Variance extracted between components	Factor correlation squared
Study 1		
perceived skill variety – perceived task variety	.77	.49
perceived skill variety – expected intrinsic work motivation	.83	.27
perceived skill variety – expected positive affect	.67	.37
Study 2		
perceived skill variety – perceived task variety	.75	.49
perceived skill variety – expected positive affect	.58	.32

3 Table. Regression coefficients, standard errors, and model summary for model including interaction effects.

	(1) DV: Expected job satisfaction		(2) DV: Expected intrinsic work motivation		(3) DV: Expected subjective performance		(4) DV: Expected positive affect		(5) DV: Expected negative affect	
Predictor	*b* (*SE*)	95% CI	*b* (*SE*)	95% CI	*b* (*SE*)	95% CI	*b* (*SE*)	95% CI	*b* (*SE*)	95% CI
Constant	1.05 (1.72)	[–2.34, 4.44]	3.19 (2.63)	[–2.00, 8.39]	2.53 (2.77)	[–2.95, 8.01]	1.34 (0.86)	[–0.36, 3.05]	1.16 (0.85)	[–0.53, 2.84]
Task rotation	–0.19 (0.27)	[–0.71, 0.33]	0.19 (0.41)	[–0.61, 0.99]	–0.46 (0.43)	[–1.31, 0.39]	–0.10 (0.13)	[–0.36, 0.17]	0.11 (0.13)	[–0.15, 0.37]
Perceived task variety	0.44 (0.98)	[–1.49, 2.37]	–1.48 (1.50)	[–4.43, 1.48]	–1.62 (1.58)	[–4.74, 1.50]	–0.28 (0.49)	[–1.25, 0.70]	0.67 (0.49)	[–0.29, 1.63]
Perceived skill variety	–0.22 (0.87)	[–1.94, 1.51]	1.16 (1.33)	[–1.48, 3.80]	1.14 (1.41)	[–1.64, 3.93]	0.29 (0.44)	[–0.58, 1.16]	–0.15 (0.43)	[–1.00, 0.71]
Perceived task identity	0.24 (0.09)	[0.06, 0.43]	–0.05 (0.14)	[–0.32, 0.23]	0.44 (0.15)	[0.14, 0.73]	0.11 (0.05)	[0.01, 0.20]	–0.11 (0.05)	[–0.20, –0.02]
Expected satisfaction of need for competence	0.46 (0.15)	[0.17, 0.76]	0.19 (0.23)	[–0.26, 0.64]	0.59 (0.24)	[0.12, 1.07]	0.19 (0.07)	[0.04, 0.33]	–0.24 (0.07)	[–0.38, –0.09]
Openness	–0.35 (0.46)	[–1.26, 0.56]	–0.42 (0.71)	[–1.82, 0.98]	–0.54 (0.75)	[–2.02, 0.94]	–0.23 (0.23)	[–0.49, 0.23]	0.46 (0.23)	[0.01, 0.91]
Task variety x openness	–0.07 (0.25)	[–0.57, 0.42]	0.43 (0.39)	[–0.33, 1.19]	0.53 (0.41)	[–0.27, 1.34]	0.08 (0.13)	[–0.17, 0.33]	–0.14 (0.13)	[–0.39, 0.11]
Skill variety x openness	0.14 (0.23)	[–0.30, 0.59]	–0.17 (0.34)	[–0.85, 0.51]	–0.23 (0.36)	[–0.95, 0.49]	0.00 (0.11)	[–0.22, 0.23]	–0.01 (0.11)	[–0.23, 0.21]
	$R^2 = .26$		$R^2 = .13$		$R^2 = .21$		$R^2 = .32$		$R^2 = .18$	
	$F(8,150) = 6.42, p < .001$		$F(8,150) = 2.80, p = .01$		$F(8,150) = 5.05, p < .001$		$F(8,150) = 8.95, p < .001$		$F(8,150) = 3.98, p < .001$	

N = 159. Unstandardized regression coefficients are reported. DV = dependent variable; CI = confidence interval.

4 Table. Indices of moderated mediation.

	(1) DV: Expected job satisfaction			(2) DV: Expected intrinsic work motivation			(3) DV: Expected subjective performance			(4) DV: Expected positive affect			(5) DV: Expected negative affect		
Indirect effect	Index	Boot *SE*	95% CI	Index	Boot *SE*	95% CI	Index	Boot *SE*	95% CI	Index	Boot *SE*	95% CI	Index	Boot *SE*	95% CI
TR → perceived task variety→ DV	–0.07	0.30	[–0.78, 0.40]	0.40	0.34	[–0.38, 0.99]	0.50	0.43	[–0.43, 1.30]	0.08	0.13	[–0.19, 0.34]	–0.13	0.16	[–0.36, 0.23]
TR → perceived skill variety → DV	0.08	0.14	[–0.17, 0.40]	–0.09	0.18	[–0.43, 0.29]	–0.12	0.24	[–0.64, 0.34]	0.00	0.05	[–0.10, 0.12]	–0.01	0.07	[–0.19, 0.11]

N = 159. Unstandardized regression coefficients are reported. DV = dependent variable; Index = index of moderated mediation; Boot *SE* = bootstrapped standard errors; CI = 95% percentile bootstrap confidence interval.
